# Uterine perforation – 5-year experience in 3-D image guided gynaecological brachytherapy at Institute of Oncology Ljubljana

**DOI:** 10.2478/raon-2013-0030

**Published:** 2013-05-21

**Authors:** Barbara Segedin, Jasenka Gugic, Primoz Petric

**Affiliations:** 1 Institute of Oncology Ljubljana, Department of Radiotherapy, Ljubljana, Slovenia; 2 National Center for Cancer care and Research Doha, Qatar

**Keywords:** uterine perforation, brachytherapy, 3D imaging, ultrasound guidance

## Abstract

**Background:**

Accurate applicator placement is a precondition for the success of gynaecological brachytherapy (BT). Unrecognized uterine perforation can lead to bleeding, infection, high doses to pelvic organs and underdosage of the target volume, resulting in acute morbidity, long-term complications and reduced chance of cure. We aimed to assess the incidence and clinical characteristics of our cases with uterine perforation, review their management and impact on the treatment course.

**Patinets and methods.:**

In all patients, treated with utero-vaginal image guided BT for gynaecological cancer between January 2006 and December 2011, the CT/MR images with the applicator in place were reviewed. The incidence of uterine perforations was recorded. Clinical factors that may have predisposed to increased risk of perforation were recorded. Management of perforations and their impact on treatment course was assessed.

**Results:**

219 patients (428 applications) were suitable for analysis. Uterine perforation was found in 13 (3.0%) applications in 10 (4.6%) patients. The most frequent perforation site was posterior uterine wall (n = 9), followed by anterior wall (n = 2) and fundus (n = 2). All cases were managed conservatively, without complications. Prophylactic antibiotics were administered in 8 cases. In 4 patients, abdominal and/or transrectal ultrasound (US) guidance was used on subsequent applications for applicator insertion; adequate applicator placement was achieved and treatment completed as planned in all cases.

**Conclusions:**

3D imaging for BT planning enables accurate identification of uterine perforations. The incidence of perforations at our department is one of the lowest reported in the literature. US guidance of applicator insertion is useful and feasible, allowing to complete the planned treatment even in challenging cases.

## Introduction

Combination of external beam radiotherapy, concomitant chemotherapy and brachytherapy (BT), is an essential treatment modality for locally advanced gynaecological cancer. In intracavitary (IC) BT of cervix cancer, an applicator (uterine tandem and vaginal ovoids, cylinder or ring) is placed in the uterine cavity and vaginal fornices in general or spinal anaesthesia. In the definitive treatment of endometrial cancer, Heyman capsules are inserted into the uterus. Intraoperative complications of IC BT include vaginal lacerations, penetration of the tandem into the uterine wall and perforation of the uterus and other pelvic organs with the applicator.^1^–^3^ Reported incidence of uterine perforations by an IC BT applicator ranges from 1.75% to 13.7%.^1^,^4^–^6^ The most common site of perforations, cited in the literature, is uterine fundus.^4^ Patients over 60 years are particularly predisposed to uterine perforation, probably due to vaginal atrophy and anatomical distortion of the cervix.^2^,^3^,^6^ Uterine perforation occurs more frequently in patients with anatomical distortions of the cervix and/or cervical stenosis due to advanced disease, post irradiation fibrosis and previous cone biopsy.^1^,^3^,^4^ Other predisposing factors include retroverted/retroflexed and extremely anteverted/anteflexed uterus.^6^–^8^

Most cases of uterine perforations resolve without squeals after conservative treatment. Nevertheless, infection, haemorrhage or peritoneal tumour-cell seeding may occur.^1^,^9^ Uterine perforations therefore require specific attention from the medical team, including the radiation oncologist, gynaecologist and anaesthesiologist. An important potential consequence of an unrecognized uterine perforation is a high level of uncertainty regarding the dose distribution in the tissues around the incorrectly inserted IC applicator. Accurate positioning of the IC applicator is critical for delivering appropriate doses of irradiation to the target volume while keeping the doses to the surrounding organs at risk below their tolerance limits. Consequently, unrecognized uterine perforation can lead to under dosage of the target volume, compromising local control probability.^10^ In addition, the perforating applicator may come in direct contact or in vicinity of the organs at risk, leading to their exposure to excessive doses, resulting in acute and long-term gastrointestinal and genitourinary complications.

At the Institute of Oncology Ljubljana, we have been using 3D MRI/CT - assisted BT for gynaecological malignancies since 2006.^11^–^18^ The main advantages of this approach over conventional 2D radiography-based techniques^19^ include: (1) the ability for individualized treatment tailoring, based on patients anatomy, tumour size and topography, (2) analysis of the dose volume histogram parameters and (3) straightforward recognition of applicator misplacement, including the uterine perforations.

The main objective of the present study was to determine the incidence and characteristics of uterine perforations at our department between January 2006 and December 2011 in the era of 3D MRI/CT – assisted BT. We evaluated the characteristics of the patients presenting with perforation and reviewed their management and impact of the perforations on the treatment course.

## Patients and methods

### Patients

All patients, treated with utero-vaginal 3D image based BT at our department for gynaecological cancer between January 2006 and December 2011, were included in analysis, irrespective of the treatment intent (curative *vs.* palliative). Pelvic MRI and/or CT images from each insertion with the applicator in place were reviewed retrospectively to find the incidence and location of uterine perforations. Patients’ medical records were studied to evaluate their management and assess the impact on the treatment course. Clinical factors, known to predispose to an increased risk of perforation, were recorded.

### Treatment

Our general treatment approach, as applied in the cases of radiotherapy with curative intent, was described elsewhere in detail.^11^–^15^ In palliative treatments, judicious individualisation of treatment techniques and dose fractionation schedules was applied. However, the brachytherapy technique did not differ considerably from the method used in curative treatment, as described below.

3D conformal CT based external beam radiotherapy (EBRT) was delivered at a 15 MV linear accelerator with a 4-field box technique, planning to apply a total dose of 45 to 50.4 Gy (1.8–2 Gy per fraction, five fractions per week) to the whole pelvis. Prophylactic paraaortic or inguinal irradiation was planned in selected patients. A boost to bring the total EBRT dose up to 60–65 Gy was planned for macroscopically involved metastatic lymph nodes. Concomitant chemotherapy (weekly cisplatin, 40 mg/m^2^) was considered in all cervix cancer patients. Following EBRT, 1–3 BT applicator insertions were scheduled in weeks 6 and 7 of treatment. Prior to each insertion, gynaecological examination was carried out by the radiation oncologist. Application technique was individually adapted to each patient at each insertion, based on the clinical and radiological findings. The insertion was carried out with the patient in subarachnoid, general or paracervical anaeasthesia, placed in lithotomy position. All applications were performed by a radiation oncologist alone, except in 3 cases, where the procedure was carried out in cooperation with a gynaecologic oncologist. Following dilatation of the cervical canal, a CT or MRI-compatible applicator was inserted into the uterus and vaginal fornices. In cervix cancer patients, a plastic or metallic tandem was used as the intrauterine part. In endometrial cancer, plastic Heyman capsules or metallic Rotte Y-applicator were applied. For other tumour sites, the decision on the intrauterine part was individual. In selected patients, a combined intracavitary/interstitial (IS) application was performed, using a ring template-cap for guidance of plastic needles into the parametria^20^–^22^ or by free-hand transvaginal insertion. Ultrasound guidance was used to aid applicator placement in challenging situations. Vaginal packing was used to fix the applicator position. Following the insertion, the patients were transported to the MRI or CT scanner. The paradigm of our BT dose prescription and treatment planning practice is represented by the image guided adaptive BT of cervix cancer and was described elsewhere.^11^–^18^

## Results

### Incidence and characteristics of perforations

From January 2006 to December 2011, 496 utero-vaginal applicator insertions were performed in 253 patients. After exclusion of 34 patients with plain radiography based BT, 428 applications in 219 patients were left for analysis.

Median patient age was 62 years (range 31 to 88), 126 (57.5%) of them were older than 60 years. The most common tumour site was cervix (172 patients; 78.5%). FIGO stage distribution of the cervix cancer patients was as follows: stage I in 21, stage II A or B in 69, stage III A or B in 67 and stage IV A in 15 cases. Thirty nine (17.8%) patients were treated for endometrial cancer (FIGO stage distribution: stage I in 14, stage II in 3, stage III in 4 and stage IV in 1 case; in 17 cases FIGO stage had not been defined). Four patients (1.8%) were treated for vaginal carcinoma, two (0.9%) for recurrent ovarian cancer with palliative intent and two (0.9%) for atypical endometrial hyperplasia.

One hundred and nineteen out of 219 patients (54.3 %) underwent 2 BT insertion procedures. The maximum number of insertions was 3 in 48 (21.9 %) patients. Tandem – ring applicator was used in 329 (76.9%), tandem – ovoids applicator in 25 (5.8%) and Heyman capsules in 74 (17.3%) applications.

**FIGURE 1:**
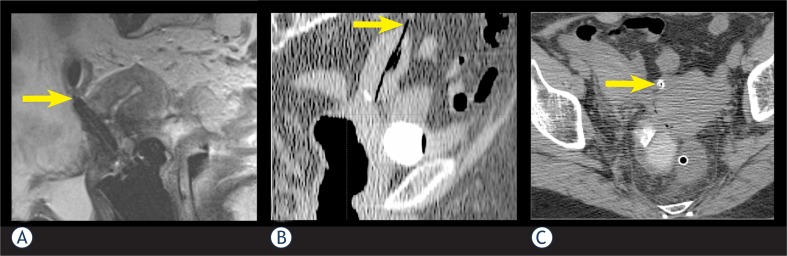
The sites of the uterine perforations. White arrows point at a perforation of posterior uterine wall (A), uterine fundus (B) and anterior uterine wall (C) by an IU applicator (tandem or Heyman capsule).

Uterine perforation was identified in 13 (3.0%) applications in 10 (4.6%) patients. Median age of patients, presenting with perforation, was 74 years (range 51–83 years). All patients with perforation, except two, were older than 60 years. Seven (70%) patients were treated for cervical cancer, two (20%) for endometrial and one (10%) for ovarian cancer. Five patients were treated with palliative intent. Posterior uterine wall was the most common site of perforation with 9 (70%) cases, followed by the anterior wall and fundus with 2 (15%) cases each (Figure 1). In most cases, one or more of the characteristics, previously described as the predisposing factors to uterine perforation (necrotic cervical tumour, cervical polyp, submucosal myoma, stenosis or distortions of cervical canal, especially due to prior conization, retroflexed or extremely anteflexed uterus) was found.^1^–^4^,^6^ The most commonly identified risk factor was age over 60 years in 8 (80%), followed by necrosis and distortion of cervical canal in 4 (40%) cases. Table 1 summarizes the frequency of these characteristics in individual patients, presenting with uterine perforation.

### Management and impact on the treatment course

In all cases where a perforation was identified on post-insertion imaging, the applicator was removed and the patient treated conservatively. General condition, blood pressure and blood count were monitored for a minimum of 24 hours. Prophylactic antibiotics, in most cases dual prophylaxis with ciprofloxacin and metronidazol, were administered in 8 (62%) patients. Three (20%) patients received blood transfusion due to a drop in haemoglobin level (median 22g/L (15–26)). All patients with uterine perforation that were treated with definitive irradiation with curative intent, completed their treatment as planned. In 4 (40%) patients, transabdominal and/or transrectal ultrasound guidance was used in subsequent application(s) and in three (30%), the subsequent application(s) were performed by a gynaecologic oncologist. At subsequent application, re-perforation occurred in three patients (without the use of ultrasound guidance) and was followed by a successful reposition of the applicator to an appropriate position under same anaesthesia in one patient. No other major intraoperative complications occurred in our series. The incidence of vaginal lacerations was not systematically recorded.

At the time of the last follow up there were no signs of intraperitoneal tumour-cell seeding in any of the patients in whom uterine perforation occurred.

## Discussion

Age over 60 years, anatomical distortion of the cervix, cervical stenosis, especially due to prior cone biopsy and variations of the physiological position of the uterus have already been reported as risk factors for uterine perforation in the literature.^1^–^4^,^6^ In our group of patients, age over 60 was the most common identified risk factor; most of the patients had at least one additional risk factor.

Corn *et al.* analysed whether the technical qualities of a BT application have an impact on the outcome of patients with locally advanced cervix cancer treated by definitive irradiation.^10^ The analysis was restricted to patients having only one intra-cavitary application to avoid potential interference effect of subsequent implants, which could compensate the presumed negative impact of a technically suboptimal first application. Plain radiographs of 66 patients were reviewed by a radiation physicist and a radiation oncologist with expertise in gynaecologic radiotherapy. On the basis of assessment of four parameters, the applications were scored as »ideal«, »unacceptable« or »adequate«. Significantly improved 5-year local control was seen when comparing ideal and adequate placement to unacceptable placement (68% *vs.* 34%, p = 0.02). A strong trend toward improved 5-year survival was also noted among the group with ideal and adequate placement as opposed to unacceptable placement (61% *vs.* 42%, p = 0.13). This study demonstrates direct influence of competent technical implant performance on local tumour control and even on survival.

Insertion of the intrauterine tandem or Heyman capsules at the time of IC BT procedure has traditionally been performed by relying only on the experience of the operating physician, the clinical assessment of the insertion adequacy and the postoperative 2D imaging – plain radiography. Specific radiological criteria, based on assessment of two orthogonal pelvic radiographs with the applicator in place were proposed to identify cases at risk of a misplaced applicator.^7^ However, notwithstanding the importance of clinical evaluation and the experience accumulated with conventional radiography-based techniques, this approach has some inherent limitations. Conventional radiography can only show the applicator position relative to the bony structures, not to the adjacent organs, even the uterus itself. Consequently, it can be expected that the techniques that employ only the clinical evaluation and plain radiography may underestimate the incidence of uterine perforations. Accordingly, the published results on the use of postoperative plain radiography show that even in case where tandem position is considered ideal according to 2D radiological criteria, 3D imaging reveals uterine perforation in approximately 3–10%.^23^,^24^ Recent review of medical records of the patients treated at our department from 2001 to 2005 with IC +/− IS BT for gynaecological tumours revealed only 1 clinically suspicious perforation (unpublished results). We can assume that this underestimates the true incidence of uterine perforations at our department during that period when only conventional 2-D radiography was used for BT planning.

Over past decades a variety of techniques have been described to determine the position of the tandem and to identify uterine perforation. Pelvic pneumography was proposed, but was soon abandoned, because it was technically too demanding.^25^ Hysterography with contrast application through a modified tandem was proposed, but fell out of favour due to the potential risk of dissemination of tumour cells into the pelvic cavity.^26^ Matsuyama *et al*. proposed attachment of surgical haemostatic clips in the uterine serosa during laparotomy in order to visualise the outer contour of the uterus on plain radiographs.^5^ The need for surgery and also the possibility to underestimate the incidence of uterine perforation led to the abandonment of the technique.^4^ Laparoscopic evaluation is also used, but it is now reserved only for rare cases in which 3D imaging techniques are not suitable or do not give enough information about tandem position.^7^

3D imaging techniques are superior to the above listed approaches, because they allow for a direct, non-invasive and unequivocal visual assessment of the tandem position and identification of eventual uterine perforation. They allow for an excellent visualization of the tandem and surrounding structures and also the relationship of the tandem to these structures. These imaging techniques are performed post-insertion with the main purpose of 3D optimization of the dose distribution. Detection of the uterine perforation by using CT or MRI requires removal of the tandem and another attempt of insertion, which consequently delays the treatment and requires anaesthesia. If the procedure is conducted in spinal anaesthesia, it is in some cases possible to correct the position of the tandem in the same anaesthesia session. Repeated imaging (CT/ MRI) is required for verification of applicator position and treatment planning, delaying the procedure and increasing the cost.

Recently, 3D image guided BT has been implemented at a growing number of institutions worldwide, tailoring the dose distribution individually, according to the patient’s patho-anatomical situation. Favourable reports on dosimetric outcome of the 3D techniques are reflected in encouraging clinical results.^13^,^27^–^32^ However, to our knowledge, the value of 3D imaging in identifying the cases of uterine perforations in BT has not been addressed so far. Uterine perforation was found in 13 (3.0%) applications in our series. By using 3D imaging, these cases were identified immediately following applicator insertion, allowing taking appropriate medical measures on time. In this way, the risk of potentially serious sequels of the perforation could be avoided in all patients and curative treatment could be completed as planned.

Ultrasound allows for a good visualization of the intrauterine applicator, uterus and urinary bladder, and can, consequently, demonstrate uterine or urinary bladder perforation. Importantly, ultrasound can be performed either postoperatively or intraoperatively as on-line guidance of the insertion.^4^,^33^–^35^ Granai *et al*. reported on the use of postoperative ultrasound in 50 consecutive applications (28 patients). In 17 applications (34%), the tandem was found to be suboptimally positioned. In 12 (24%) applications, it penetrated the myometrium and in 5 (10%) cases it frankly perforated the uterine wall. In all cases where postoperative ultrasound identified the applicator malposition, clinical and radiographic assessment indicated accurate placement. In a report on the use of intraoperative ultrasound in 73 consecutive applications, ultrasound allowed direct visualization of the procedure and accurate tandem positioning in 72 cases.^4^ Consistent with findings of other authors, intraoperative transabdominal ultrasound was identified as a useful adjunct at the time of IC BT, allowing to complete the planned treatment even in difficult cases.^35^,^36^ According to Davidson *et al.*, the use of transabdominal US influenced the length and angle of IU tandem chosen in 49% of patients.^24^ They also report on shortening of the applicator insertion time and fewer requests for assistance of a gynaecologist at the procedure. Fleischer *et al*. reported on use of transrectal sonography (TRUS) at the time of IC BT and also for some other gynaecological procedures.^37^ They found transrectal ultrasound to be useful in providing guidance for these procedures and at the same time helpful in avoiding uterine perforation and urinary bladder injury. Sharma *et al.* published a series of 40 gynecological BT procedures performed with TRUS guidance.^38^ In all cases, adequate position of IU tandem was achieved, while the procedure time was not prolonged with the use of TRUS. In our experience, the use of intraoperative US provides immediate feedback information and enables a guided application. At our department, intraoperative, mainly transrectal ultrasound guidance of insertion is used in challenging cases. In the present study, transabdominal and/or transrectal ultrasound was used in 4 (31%) patients with prior uterine perforation, and enabled successful insertion in all cases. Importantly, corrective measures can be made immediately and therefore there is no need for an additional anaesthesia. Moreover, ultrasound is widely available, feasible and associated with low costs compared to other imaging modalities.

## Conclusions

3D imaging techniques, used for BT planning, not only allow for the treatment optimisation, but also enable accurate identification of uterine perforations, preventing potential acute and chronic complications, including treatment failure due to underdosage of the tumour and overdosage of the organs at risk. Transrectal or transabdominal intraoperative ultrasound guidance is a very useful tool at the time of IC BT and generally allows for an appropriate positioning of the applicator, even in difficult cases. Special care is warranted when performing BT applications in elderly patients in the presence of known predisposing factors for uterine perforation. In such cases, as well as in patients with prior perforation, US guidance is recommended.

## Figures and Tables

**TABLE 1:** Presence of the known risk factors in individual patients and tumours in which uterine perforation occurred

**Risk factor**	**Patient presenting with uterine perforation**									
	1	2	3	4	5	6	7	8	9	10
Age > 60 y	•	•	•	•	•		•	•	•	
Necrosis			•			•		•		•
Cervical polyp					•					
Myoma		•							•	
Distorted CC		•	•	•		•		•		
RF uterus				•				•	•	
Conization				•						

y = years; CC = cervical canal; RF = retroflected
